# Geographic Variation Characteristics of Endophytic Bacterial Communities in Roots of *Hippophae rhamnoides* subsp. *sinensis* Rousi in the Arid Region of Northwest China

**DOI:** 10.3390/microorganisms14081829

**Published:** 2026-08-19

**Authors:** Hongyuan San, Pei Gao, Siyu Guo, Guisheng Ye, Yuhua Ma, Liyan Zhao, Ruisi Ni, Yufeng Zhang, Liping Ma

**Affiliations:** 1 College of Forestry and Grass, Qinghai University, Xining 810016, China; 18997410490@163.com (H.S.); y200954000466@qhu.edu.cn (P.G.); siyu_guo1005@outlook.com (S.G.); 13119706011@163.com (L.Z.); 18797114871@163.com (R.N.); 13897301837@163.com (Y.Z.); 1389721594@163.com (L.M.); 2 College of Agriculture and Animal Husbandry, Qinghai University, Xining 810016, China; qhxjygs@163.com

**Keywords:** Qinghai–Tibet Plateau, climatic characteristics, altitude, soil physicochemical properties, microbial community structure

## Abstract

As an endemic woody plant resource widely distributed in the arid regions of northwestern China, *Hippophae rhamnoides* subsp. *sinensis* Rousi possesses both ecological and medicinal value. Elucidating the effects of climate and soil conditions on the composition, structure, and function of its root-associated bacterial communities is of practical significance for the development and utilization of these indigenous plant resources in this water-limited region. In this study, root samples of *H. rhamnoides* were collected from 12 sampling sites in the arid region of Northwest China. High-throughput amplicon sequencing was employed to examine bacterial composition, alpha and beta diversity, molecular co-occurrence networks, and PICRUSt-based functional prediction. Mantel tests and redundancy analysis (RDA) were further applied to identify what is associated with shaping bacterial community structure. The main results are summarized as follows: (1) There were significant differences in the number of ASVs among the various sampling sites, with the P8 site having the highest number (435) and the P5 site having the lowest (156). The dominant phyla in the community were Proteobacteria, Actinobacteria, and Cyanobacteria. (2) Both α and β diversity showed significant differentiation among the 12 sampling sites. The Ace and Chao1 indices of P8 sampling sites were the highest, while P5 sampling sites were the lowest. In terms of community aggregation, P7 and P12 sampling sites showed tighter clustering, whereas P4 and P5 sampling sites showed more scattered bacterial assemblages. (3) Functional prediction suggested that metabolism, environmental information processing, and genetic information processing functional potential may all be dominant across 12 sampling sites. The abundance of environmental information predicted that the processing functional potential of the P9 sampling site was higher than that of the other 11 sampling sites, while the genetic information processing functional potential was low. (4) Cluster analysis grouped the 12 sampling sites into two groups: sampling sites P10, P11, and P12 were grouped into Group 1, while the remaining 9 sampling sites were grouped into Group 2. (5) RDA revealed that altitude was associated with shaping the root endophytic bacterial community structure of *H. rhamnoides*, followed by soil total nitrogen. Taken together, the *H. rhamnoides* populations investigated and sampled in this study were predominantly distributed in neutral-to-alkaline arid soils. The relevant environmental factors are significantly correlated with the alpha diversity patterns of root endophytic bacteria of this species, and they can modulate the community assembly processes, functional allocation characteristics, and co-occurrence network structures of these root endophytic bacteria.

## 1. Introduction

In the arid area of northwest China, the spatial differentiation of climate patterns and soil properties is associated with the reorganization of the *Hippophae rhamnoides* subsp. *sinensis* Rousi community in China, which leads to the response of the endophytic bacteria community structure in the root system [1,2,3]. The community characteristics of plants and their root endophytic microorganisms represent not only adaptive responses to environmental conditions but also reflect the directional adjustment of life-history strategies for both components under environmental change, holding profound implications for the maintenance of ecosystem functions [1]. Previous studies have provided some evidence on this issue. For example, the structural variation of root endophytic fungal communities in *H. rhamnoides* is primarily regulated by regional electrical conductivity and altitude [3]; analyses of the rhizosphere soil microorganisms of *H. rhamnoides* have shown that altitude and soil water content are the core environmental factors driving the differentiation of rhizosphere bacterial community composition and structure [4]; field investigations across different habitats in the arid region of Northwest China have further confirmed that the combined effects of soil nutrients, moisture conditions, and salinity–alkalinity properties collectively govern the spatial variation patterns of rhizosphere microbial communities of local *Kengyilia thoroldiana* [5]. These results suggest that the structure of plant roots and their endophytic bacteria communities is an ideal starting point for understanding the interaction between plants and the environment [4,5]. Among them, ASV number, α/β diversity, and dominant bacteria composition are highly variable and easy to obtain, which is suitable as a breakthrough to analyze the relationship between microbial community structure and environmental factors [6].

*H. rhamnoides*, also known as “sour thorn” or “black thorn,” belongs to the genus *Hippophae* in the family Elaeagnaceae. It is a deciduous shrub or tree primarily distributed in China, Nepal, and Bhutan [4,7]. Within the territory of China, it is mainly distributed in Qinghai, Gansu, and Xinjiang, with sporadic distribution in Sichuan, Xizang, and Hebei. This species grows at elevations ranging from 1200 to 4800 m and is commonly found on sun-exposed ridges, in valleys, on dry riverbeds, and on gravelly or sandy slopes. It is characterized by its cold tolerance, drought tolerance, and resistance to radiation, making it an important tree species for ecological restoration in arid regions [4]. Furthermore, *H. rhamnoides* has a well-developed root system that can spread laterally to form a sand-stabilizing network, playing a key role in maintaining surface stability in arid regions. It is regarded as a core native tree species for the construction of regional ecological security barriers [8].

Up to now, the research on endophytic bacteria in the roots of other plants in the arid area of northwest China has accumulated to some extent, but the systematic research on the community structure of endophytic bacteria in the roots of *H. rhamnoides* is still blank [3,4,7]. In order to fill this gap, this study took 12 natural *H. rhamnoides* populations in the northwest arid area of China as the object, combined with Illumina MiSeq sequencing, soil physical and chemical properties determination, and climate data collection; analyzed the differentiation law of endophytic bacteria community structure and function in their roots; and identified the key environmental factors driving community variation. This study aims to provide a microbiological theoretical basis for the application of *H. rhamnoides* in vegetation restoration and ecological barrier construction in the arid region of Northwest China, promoting a shift in research perspective from single-plant traits toward the synergistic mechanisms between ecology and microorganisms.

## 2. Materials and Methods

### 2.1. Overview of the Distribution and Climatic Characteristics of 12 H. rhamnoides Sampling Sites

**Figure 1:**
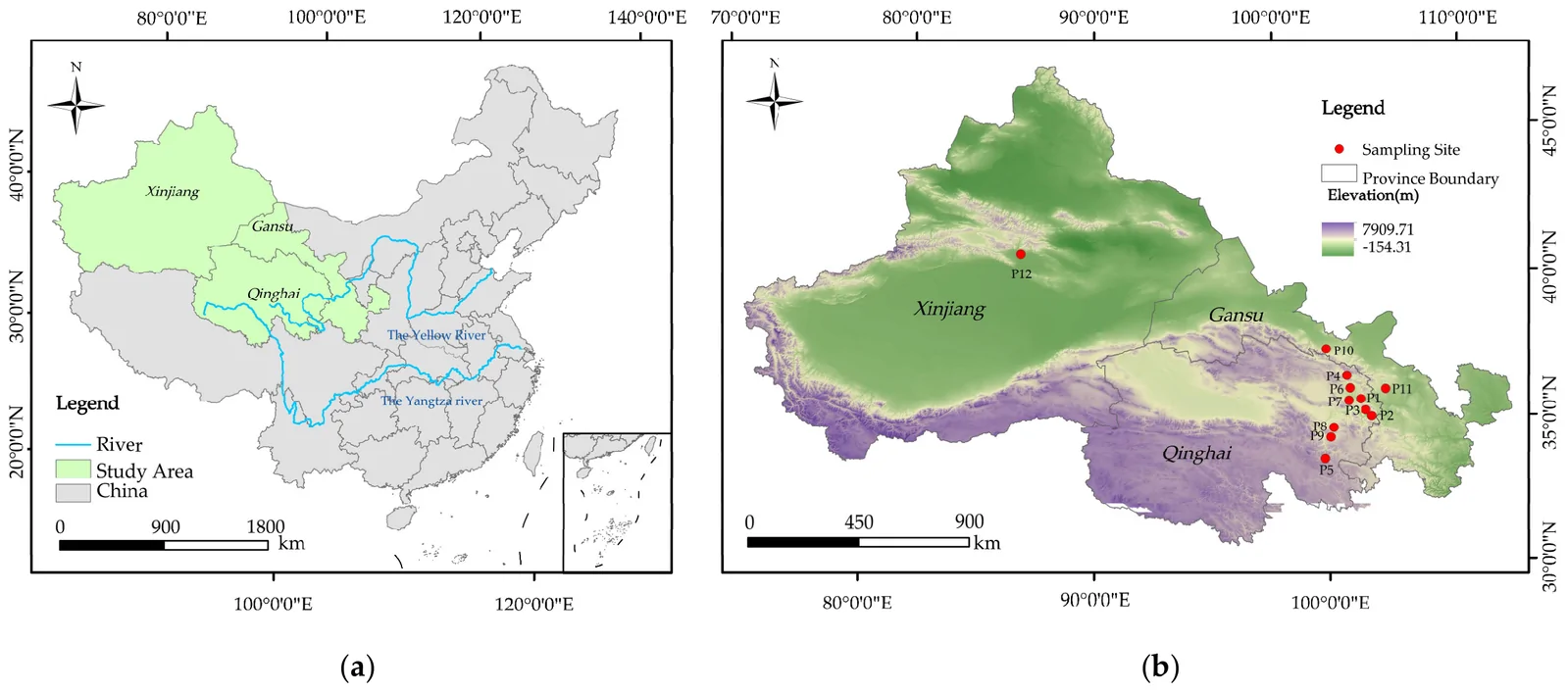
Spatial distribution of sampling sites across the 12 distribution areas of *H. rhamnoides*. Notes: (**a**) Map of China; (**b**) detailed local map of the sampling sites in the study area across Qinghai, Gansu, and Xinjiang provinces. This map was created based on the official standard base map obtained from the Standard Map Service website of the China Bureau of Surveying, Mapping, and Geoinformation, with the base map approval number GS (Beijing) No. 0650 (2024). Calculations show that the pairwise distances between all sampling sites range from 34.28 to 15,817.74 km.

From mid-July to early August 2023, a total of 12 sample plots (sampling sites) were established across three provinces (regions) of Qinghai, Gansu, and Xinjiang, China. Specifically, Qinghai Province comprised nine sampling sites: Ping’an District (P1), Xunhua Salar Autonomous County (P2), Hualong Hui Autonomous County (P3), Menyuan Hui Autonomous County (P4), Maqin County (P5), Datong Hui and Tu Autonomous County (P6), Huangzhong District (P7), Guinan County (P8), and Tongde County (P9). Gansu Province included two sampling sites: Minle County (P10) and Yongdeng County (P11). The Xinjiang Uygur Autonomous Region had one sampling site: Hejing County (P12). The spatial distribution of all sampling locations is illustrated in Figure 1, with detailed geographic information and climatic parameters summarized in Supplementary Table S1.

All of the above regions have a highland continental climate, characterized by generally low precipitation (annual average of 30–140 mm), relatively low temperatures (annual average of −2.0–12.0 °C, with extreme lows reaching −25.5 °C), high evaporation rates (annual average of 1200–2500 mm), abundant sunshine (annual average of 2100–2800 h), and significant diurnal temperature variations (reaching up to 20–25 °C) [9]. Common accompanying plants in *H. rhamnoides* habitats include *Dasiphora fruticosa*, *Artemisia hedinii* Ostenf, *Artemisia frigida* Willd, *Poa araratica* Trautv, *Poa pratensis* L., *Festuca sinensis*, and *Aconitum pendulum*, among others.

### 2.2. Collection of H. rhamnoides Root Samples and Rhizosphere Soil Samples

Within the 12 wild *H. rhamnoides* sampling sites, nine replicate sampling units were systematically arranged along an S-shaped sampling transect at each sampling site, with a constant spacing of 8 m between adjacent units. We selected *H. rhamnoides* plants at the fruit-bearing stage, with an age ranging from 5 to 10 years. All sampled *H. rhamnoides* plants exhibited highly similar genotypes. However, due to habitat differences, genetic variation exists among populations, including mutations, chromosomal recombination, and allelic differences, resulting in non-identical genotypes among different populations. The combination of environmental variation and intrinsic genetic differences contributed to the unique plant morphology observed across the 12 sampling sites. The height of all sampled *H. rhamnoides* plants ranged from 2.2 to 4.2 m, crown width from 1.4 to 2.2 m, and basal diameter from 3.5 to 5.5 cm. The rhizosphere soil of *H. rhamnoides* was collected using the standardized Riley–Barber rhizosphere soil sampling method [10]. The specific procedure was as follows: first, the entire root system of *H. rhamnoides* was excavated, followed by vertical shaking to naturally detach the non-rhizosphere soil; subsequently, precise sampling was performed along the root axis using a sterile brush, systematically collecting soil particles adhering to the root surface. At each sampling site, three adjacent sampling units were selected, and equal amounts of rhizosphere soil samples were thoroughly mixed to form one composite sample; through a combined design of 12 sampling areas × 3 replicate points, a total of 36 ecologically representative rhizosphere soil sample pools were established.

All soil samples were collected from vertical soil layers at a depth range of 0–50 cm. The soil samples were passed through a standard 2 mm sieve, sealed in sterile bags, and stored at −20 °C. Soil physicochemical properties were determined strictly following the technical specifications of Soil Agro-Chemical Analysis [11], and the measured parameters included pH, electrical conductivity, water content, organic matter content, and nitrogen, phosphorus, and potassium contents. The specific determination methods for the 10 soil indicators are as follows: soil organic matter content (SOM) was determined using the potassium dichromate oxidation method; soil total nitrogen content (STN) was determined by the Kjeldahl method; soil total phosphorus content (STP) was quantitatively analyzed using the molybdenum-antimony colorimetric method; soil total potassium content (STK) was determined by flame photometry; soil available nitrogen (SAN), available phosphorus (SAP), and available potassium (SAK) contents were determined by the alkaline hydrolysis diffusion method, the molybdenum-antimony colorimetric method, and flame photometry, respectively; soil water content (SWC) was determined by the oven-drying method; and soil pH and electrical conductivity (SEC) were determined by potentiometric titration and the electrode method, respectively [3,4] (for detailed parameters, see Supplementary Table S2).

*H. rhamnoides* root samples were collected synchronously with the soil samples. During field sampling, three sterile water blank samples were carried throughout the process. After completing the rhizosphere soil sampling, fresh fine roots of healthy *H. rhamnoides* plants were collected using a sterile stainless steel spatula that had been pre-sterilized by flaming and cooled. After carefully removing nodule tissues and attached large soil particles, the root samples were immediately placed into sterile centrifuge tubes, quickly frozen in liquid nitrogen for temporary preservation, and then rapidly transported back to the laboratory. After the sample entered the laboratory, the surface of the root system was gently washed with precooled sterile flowing water to loosen the soil particles. At the same time, the same experimenter further removed the residual nodule tissue with a sterile scalpel according to the unified standard, and then the standardized surface sterilization process was completed in a clean bench. The roots were completely immersed in 75% ethanol with shaking for 30 s for disinfection, immediately transferred to 3% sodium hypochlorite solution for 3 min, and then rinsed five consecutive times with sterile water, with each rinse lasting no less than 30 s, to thoroughly remove residual disinfectant from the root surface. To verify the effectiveness of the surface sterilization, the rinse water from the final wash was collected, and 100 μL was spread onto LB solid agar plates (Solarbio Science & Technology Co., Ltd., Beijing, China). The plates were incubated in a constant-temperature incubator (Yiheng Scientific Instruments Co., Ltd., Shanghai, China) at 28 °C in the dark for 48 h. The surface sterilization was considered qualified when no bacterial colony growth was observed on the plates. After completing the above procedures, the root samples were finally transferred to a −80 °C ultra-low temperature freezer (Zhongke Duling, Hefei, China) for long-term storage, to be used for subsequent extraction and analysis of the endophytic bacterial community [3,12].

### 2.3. DNA Extraction, PCR Amplification, and High-Throughput Amplicon Sequencing of Endophytic Bacteria from H. rhamnoides Roots

Root samples of *H. rhamnoides* collected from 12 sampling sites were fully ground into a fine powder using liquid nitrogen, and 0.5 g of the resulting material was taken for DNA extraction. During DNA extraction, three blank controls were processed in parallel. The extraction was carried out following the manufacturer’s instructions for the plant DNA extraction kit (NucleoSpin; Merck, Düren, Germany), and three biological replicates were prepared for each sampling site. The concentration and purity of the extracted DNA were assessed by 1% agarose gel electrophoresis combined with a nucleic acid analyzer. The quality criteria for the submitted DNA samples were as follows: DNA concentration ≥ 50 ng/μL and volume ≥ 60 μL; DNA purity with an A260/A280 ratio of 1.8–2.0 (indicating no significant protein or RNA contamination); and agarose gel electrophoresis showing clear main bands with no degradation or restriction enzyme digestion artifacts. PCR products that passed quality control and quantification were used to construct MiSeq sequencing libraries, which were subsequently subjected to high-throughput sequencing on the Illumina MiSeq platform using the PE300 mode [4,13]. Sequencing was performed by Beijing Novogene Bioinformatics Technology Co., Ltd. (Beijing, China). The bacterial 16S rRNA gene V4 region was amplified using the universal primer pair 515F/806R (515F: 5′-GTGCCAGCMGCCGCGGTAA-3′; 806R: 5′-GGACTACHVGGGTWTCTAAT-3′) [13,14].

The PCR reaction mixture, with a total volume of 20 μL, consisted of 2 μL of 10× buffer, 2 μL of 2.5 mmol/L dNTPs, 0.8 μL each of forward and reverse primers, 0.2 μL of rTaq polymerase, 0.2 μL of BSA, and 10 ng of template DNA; the remaining volume was made up to 20 μL with ddH_2_O. The amplification program was set as follows: initial denaturation at 95 °C for 3 min; then, 35 cycles were performed, with each cycle consisting of 95 °C for 30 s, 55 °C for 30 s, and 72 °C for 45 s; followed by a final extension at 72 °C for 5 min, and then held at 4 °C [4,15]. In addition, a no-template negative control was also included during the PCR amplification. After amplification, the products that were verified to be qualified via agarose gel electrophoresis were mixed in equimolar amounts and subsequently purified.

Library construction strictly followed the standard procedures: after a second round of electrophoresis, the target bands were recovered using a Qiagen gel extraction kit (Qiagen, Düsseldorf, Germany); the libraries were prepared with the NEBNext^®^ Ultima™ II DNA Library Prep Kit (New England Biolabs, Ipswich, MA, USA) and quantified by Qubit and quantitative real-time PCR (qPCR). Qualified libraries were sequenced on the NovaSeq 6000 platform (Illumina, San Diego, CA, USA). The resulting sequencing data were subjected to quality control and chimera removal. Sequences were denoised and filtered (abundance < 5) using the DADA2 plugin in QIIME 2 software, generating amplicon sequence variants (ASVs) and a feature table. Subsequently, taxonomic assignment of ASVs was performed using QIIME 2 (version 2022.02) with the classify-sklearn algorithm and a pre-trained naive Bayes classifier against the Greengenes 13_8 database [4,16]. ASVs assigned to chloroplasts and mitochondria were removed, and the data were rarefied during the analysis to ensure consistent sequencing depth across samples. Sequencing and annotation were performed by Novogene Bioinformatics Technology Co., Ltd. (Beijing, China) [3,4].

### 2.4. Data Analysis

Raw data were organized using Microsoft Excel 2019 (Microsoft, Washington, DC, USA), and statistical analyses were performed using SPSS 25.0 (IBM, New York, NY, USA). In addition, visualization analyses of bacterial communities in *H. rhamnoides* roots were performed through the Novogene cloud platform (https://magic.novogene.com, accessed on 10 June 2026) [3,4], including abundance analysis, diversity analysis, cluster analysis, LEfSe statistical analysis, and PICRUSt functional prediction. Rarefaction curves, species relative abundance plots, PCoA plots (based on Bray–Curtis dissimilarity), and UPGMA dendrograms were generated using QIIME 2 software (version 202202), and α-diversity indices (OTUs, Shannon index, Simpson index, Pielou index, Invsimpson index, Chao1 index, ACE index, and Good’s coverage) were calculated. To further investigate the differences in community structure among grouped samples, LEfSe statistical analysis was applied to test the significance of differences in species composition and community structure [3,4]. PICRUSt software is a bioinformatics software package for predicting microbial functions based on marker genes (such as 16S rRNA). By analyzing the correlation between amplicon annotation results and the corresponding functional database (KEGG), PICRUSt software (version 2.5.1) was used at the first, second, and third levels to predict and analyze the microbial community functions in ecological samples [17]. Networkx (version 1.11) was used to calculate correlations among species in order to construct a molecular network. R software (version 3.5.2) was employed to generate correlation heatmaps between environmental factors, soil physicochemical factors, and microbial diversity, and Canoco 5.0 software (Microcomputer Power, New York, NY, USA) was used to perform redundancy analysis (RDA) on the above three factors [3,4,9].

## 3. Results

### 3.1. Variations in 16S Sequencing Quality and ASV Abundance of Endophytic Bacteria in H. rhamnoides Roots

**Figure 2:**
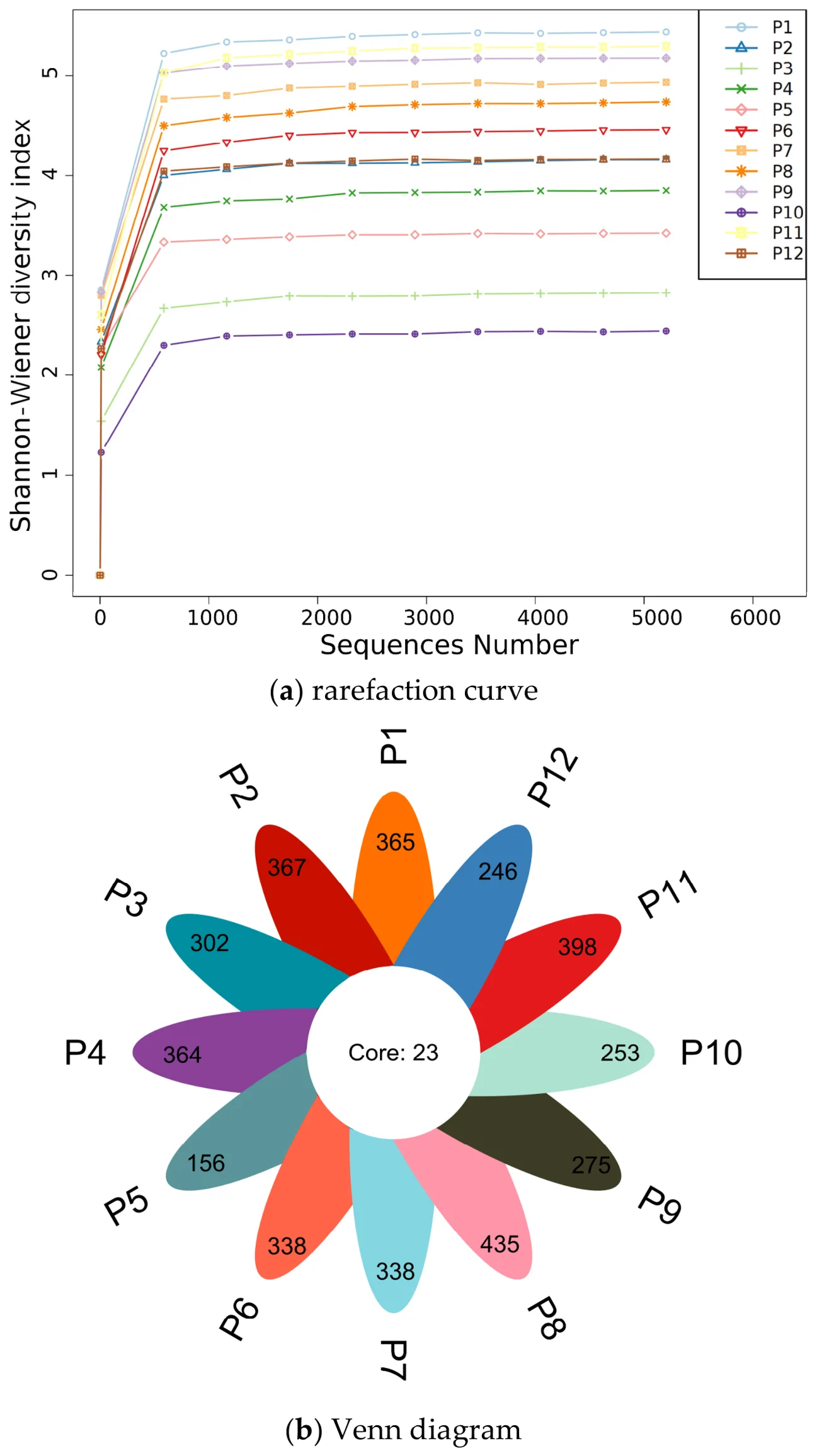
Analysis of the rarefaction curve and ASV quantity of the sampling sites. (**a**) Rarefaction curve; (**b**) Venn diagram. Note: A total of 36 samples were sequenced for root endophytic bacteria. All data in the figure are expressed as the mean values of three replicate samples at each sampling point; the same applies hereinafter.

As shown in Figure 2a, as the number of sequencing reads gradually increases; the rarefaction curves corresponding to the 12 sampling sites tend to flatten out when the number of reads reaches the 1000–1500 range. This indicates that the current sequencing depth has essentially reached saturation, covering more than 95.0% of the endophytic bacterial communities in the root systems of *H. rhamnoides*, and that the sequencing volume was set appropriately. If the dataset is further expanded to more than 1500 records, only a very small number of low-abundance species can be detected, and the marginal benefit is extremely low. Therefore, the sequencing results of the existing root samples effectively represent the true structure of the endophytic bacterial communities in the roots of the 12 sampling sites.

The distribution characteristics and quantitative relationships of shared and unique ASVs among the 12 sampling sites were visualized using a Venn diagram, with the results shown in Figure 2b. There are obvious differences in the distribution pattern of ASV among different sampling sites: the P8 sampling site has 435 ASVs, ranking first and being the only one with more than 400; the number of ASVs in the P5 sampling site is the lowest, only 156, and the number of ASVs in other species is between 200 and 400. Further analysis showed that 23 ASVs were stably detected in all 12 sampling sites, and these shared ASVs constituted the core component of the endophytic bacteria community in the *H. rhamnoides* root system.

### 3.2. Changes in the Species Composition and Relative Abundance of Endophytic Bacteria in the Root Systems of H. rhamnoides

**Figure 3:**
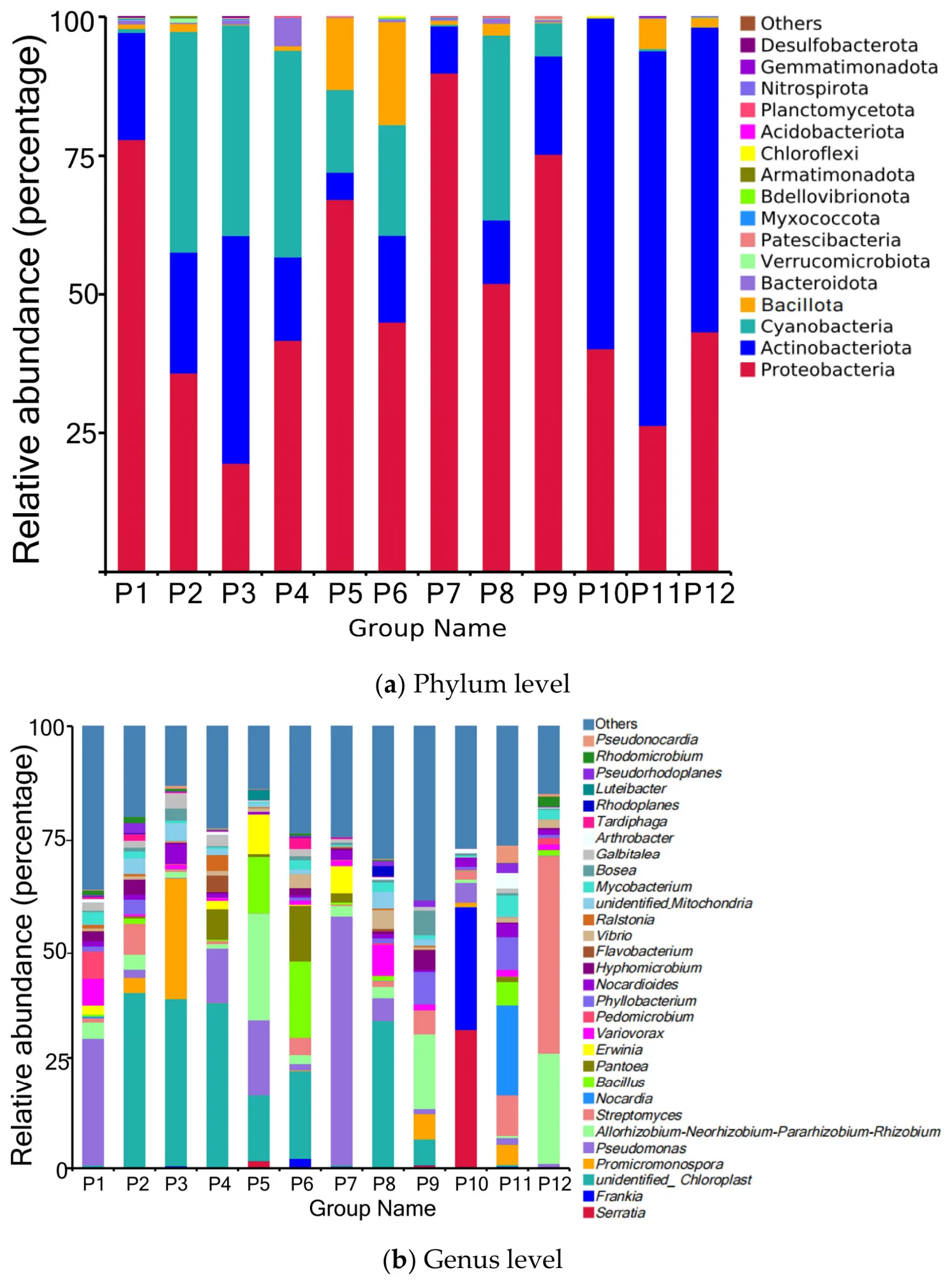
Relative abundances at the phylum and genus levels for 12 sampling sites. (**a**) Phylum level; (**b**) Genus level. Note: The data on the proportion of bacterial abundance are expressed as average values (*n* = 3).

As shown in Figure 3a, based on the single-sample statistical rule, bacterial groups with a relative abundance greater than 1% were screened from the root endophytic bacterial samples of *H. rhamnoides* across the 12 sampling sites, resulting in 16 high-abundance phyla, and their distribution characteristics are as follows. At the phylum level, the three most abundant phyla were Proteobacteria (19.60–89.96%), Actinobacteriota (4.86–67.56%), and Cyanobacteria (0.04–39.80%), followed by Bacillota (0.13–18.54%) and Bacteroidota (0.00–5.10%). There were significant differences in the composition of dominant bacterial phyla among the different sampling sites. Among them, the Proteobacteria phylum dominated at seven collection sites (P1, P4, P5, P6, P7, P8, and P9); the Actinobacteria phylum was the dominant group at P2, P10, P11, and P12; and the Cyanobacteria phylum was prominent at the P2 sampling site. Furthermore, compared with the P5 sampling site, the abundance of Actinobacteriota among root-associated bacteria increased to varying degrees in the remaining 11 sampling sites.

As shown in Figure 3b, based on the single-sample statistical rule, bacterial groups with a relative abundance greater than 0.1% were screened, resulting in 30 high-abundance genera, and their distribution characteristics are as follows. At the genus level, *Serratia* (0.00–31.39%), *Frankia* (0.00–27.78%), unidentified chloroplasts (0.06–39.82%), *Promicromonospora* (0.00–27.17%), *Pseudomonas* (0.15–56.37%), *Allorhizobium–Neorhizobium–Pararhizobium–Rhizobium* (0.47–24.99%), *Streptomyces* (0.04–44.84%), and *Nocardia* (0.00–20.52%) were the eight bacterial genera with higher abundances across the 12 sampling sites. The relative abundances of each genus showed distinct variations among different sampling sites. In addition, compared with sampling site P5, the abundances of *Promicromonospora* and *Streptomyces* among the endophytic bacteria in the root systems were higher at the remaining 11 sampling sites.

### 3.3. LEfSe Analysis of Root Endophytic Bacteria of H. rhamnoides

**Figure 4:**
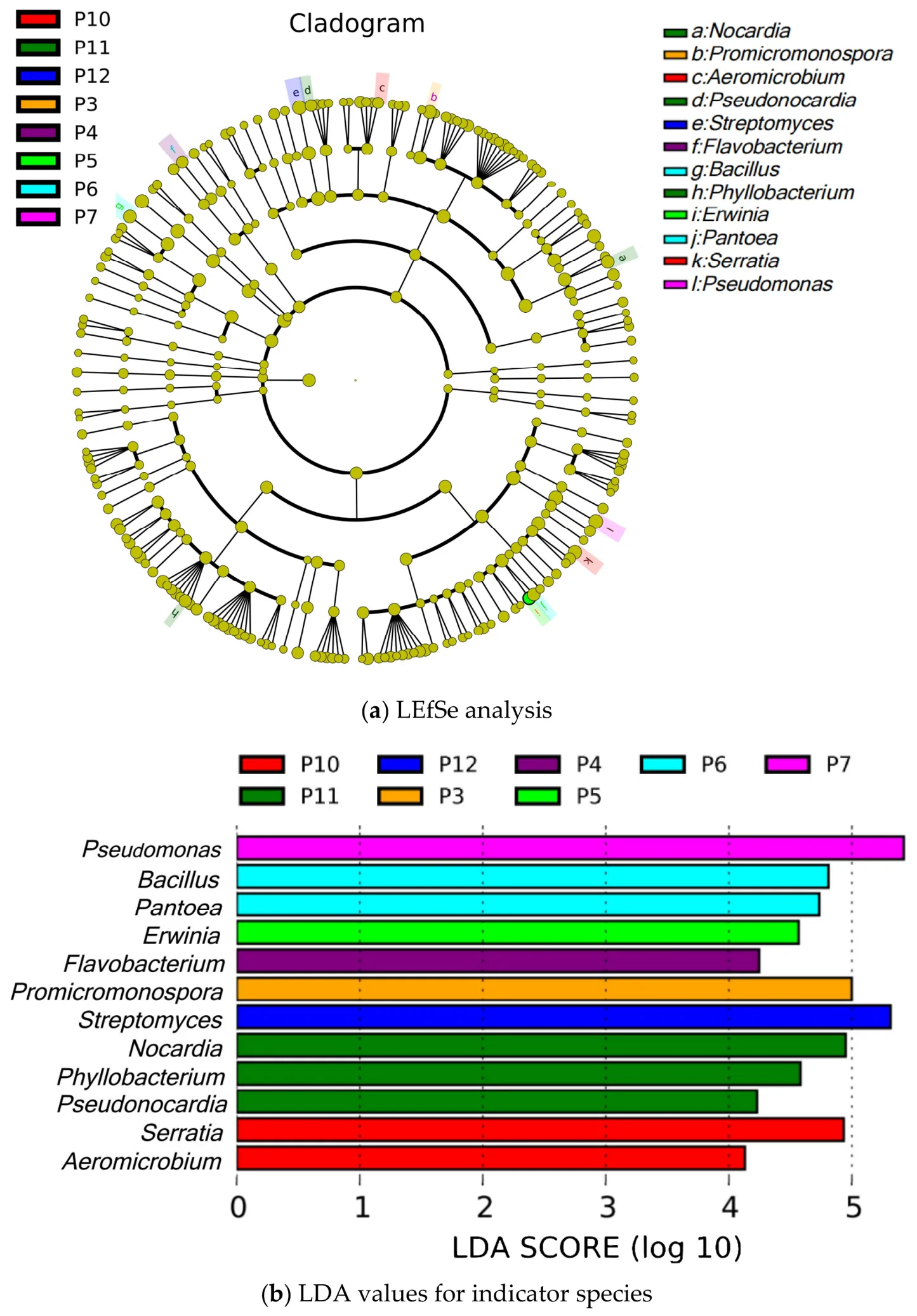
LEfSe analysis at the genus level for 12 sampling sites. (**a**) LEfSe analysis; (**b**) LDA values for indicator species. Note: LDA > 4.0.

Using LEfSe analysis, we identified the predicted indicator species that contributed most significantly to the differences in the endophytic bacterial communities of the roots of 12 *H. rhamnoides* sampling sites (LDA value > 4.0). As shown in Figure 4, a total of 12 predicted biomarkers were identified in the bacterial communities from the 12 sampling sites: one each from sampling sites P3, P4, P5, P7, and P12; two each from sampling sites P6 and P10; and three from sampling site P11. In terms of taxonomic affiliation, the markers with the highest scores for each sampling site were as follows: P3, *Promicromonospora* (LDA = 5.00); P4, *Flavobacterium* (LDA = 4.25); P5, *Erwinia* (LDA = 4.57); for sampling site P6, *Bacillus* (LDA = 4.81); for sampling site P7, *Pseudomonas* (LDA = 5.42); for sampling site P10, *Serratia* (LDA = 4.93); for sampling site P11, *Nocardia* (LDA = 4.95); and for sampling site P12, Streptomyces (LDA = 5.31).

### 3.4. Analysis of Bacterial Community Diversity in the Root Systems of H. rhamnoides

#### 3.4.1. Analysis of α-Diversity in the Bacterial Communities of *H. rhamnoides* Root Systems

**Figure 5:**
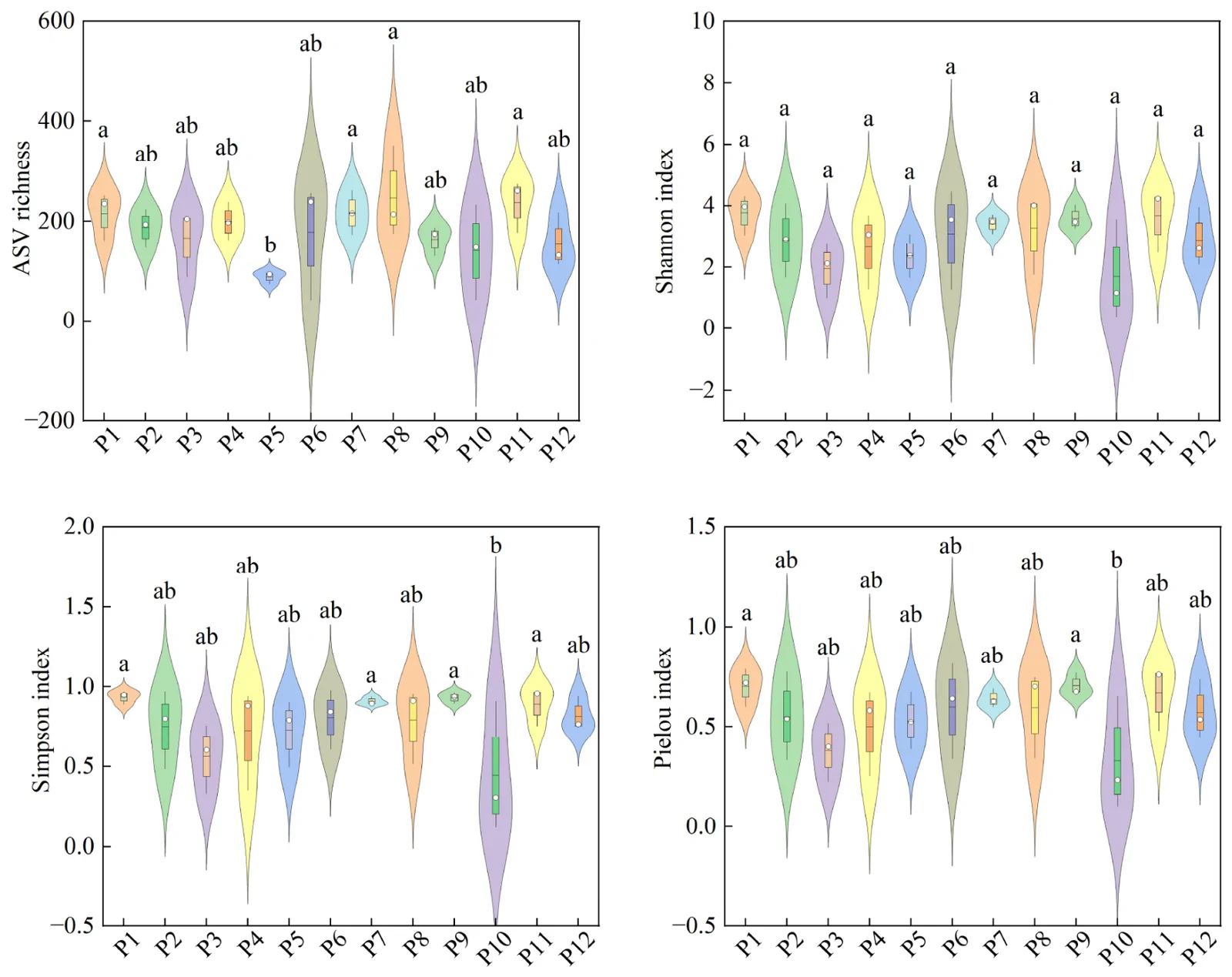
Alpha diversity of bacterial communities from 12 sampling sites (ASV richness, Shannon index, Simpson index, Pielou index). Note: Different lowercase letters indicate significant differences at the *p* < 0.05 level.

As shown in Figure 5, the α diversity index of the endophytic bacterial community in the root system of *H. rhamnoides* from 12 sampling sites showed obvious differences among sampling sites. In terms of the number of ASVs, the P8 sampling site was the highest (246.67), which was 179.26% higher than the P5 sampling site, with a significant difference (*p* < 0.05). The Shannon index of the P1 sampling site was the highest (3.78), which was 122.35% higher than that of the P10 sampling site, but the difference was not significant (*p* > 0.05). The Simpson index is arranged in the order of P1 > P9 > P7 > P11 > P12 > P6 > P8 > P2 > P5 > P4 > P3 > P10, among which P1, P7, P9, and P11 have no significant difference (*p* > 0.05), but they are significantly lower than those of P10 (*p* < 0.05). Regarding the Pielou index, sampling site P9 had the highest value (0.71), which was 115.15% higher than that of sampling site P10 (*p* < 0.05).

As illustrated in Supplementary Figure S1, the Invsimpson index was highest at sampling site P11 (20.30), approximately 7.46 times that of sampling site P3. Both the Ace and Chao1 indices peaked at sampling site P8, with values of 273.44 and 271.16, respectively, representing increases of 178.91% and 183.70% compared to sampling site P5 (*p* < 0.05). Goods coverage was highest at the P5 sampling site (0.998), slightly higher than at the P4 sampling site (an increase of 0.76%). In summary, compared to the P5 sampling site, the P8 sampling site significantly increased the number of ASVs and diversity indices such as Ace and Chao1 in the endophytic bacteria of *H. rhamnoides* roots.

#### 3.4.2. Analysis of β-Diversity (PCoA) of the Endophytic Bacterial Community in *H. rhamnoides* Roots

PCoA analysis based on Bray–Curtis distances revealed the within-group dispersion of endophytic bacterial communities in roots of *H. rhamnoides* from 12 sampling sites (Supplementary Figure S2). The results showed that the bacterial communities at sampling sites P7 and P12 were most tightly clustered, with the smallest within-group dispersion; the other ten sampling sites exhibited relatively weaker within-group homogeneity, especially sites P4 and P5, which had the lowest degree of clustering. The first two principal coordinates (PC1 and PC2) explained 18.46% and 10.76% of the variation, respectively, with a cumulative explanation of 29.22%, indicating relatively high between-group differences.

### 3.5. Co-Occurrence Molecular Network Variation of Endophytic Bacteria in H. rhamnoides Roots

To elucidate the interspecies interactions of endophytic bacteria in the roots of *H. rhamnoides* from 12 sampling sites at the genus level, a genus-level co-occurrence molecular network was constructed in this study (Supplementary Figure S3). The network comprised 506 nodes and 1812 edges, with an average degree of 7.16, an average path length of 3.71, a network diameter of 8.11, a network density of 0.014, and a clustering coefficient of 0.58. The edge connections were predominantly positive correlations. Among them, Proteobacteria (53.36%) and Actinobacteriota (38.54%) ranked first in both degree and closeness centrality, occupying the core positions in the network; Bacillota (2.96%), Cyanobacteria (1.98%), and Bacteroidota (1.38%) had lesser influence. These bacteria could establish extensive interactions with other bacteria.

### 3.6. Functional Prediction of Root Endophytic Bacterial Communities in H. rhamnoides

**Figure 6:**
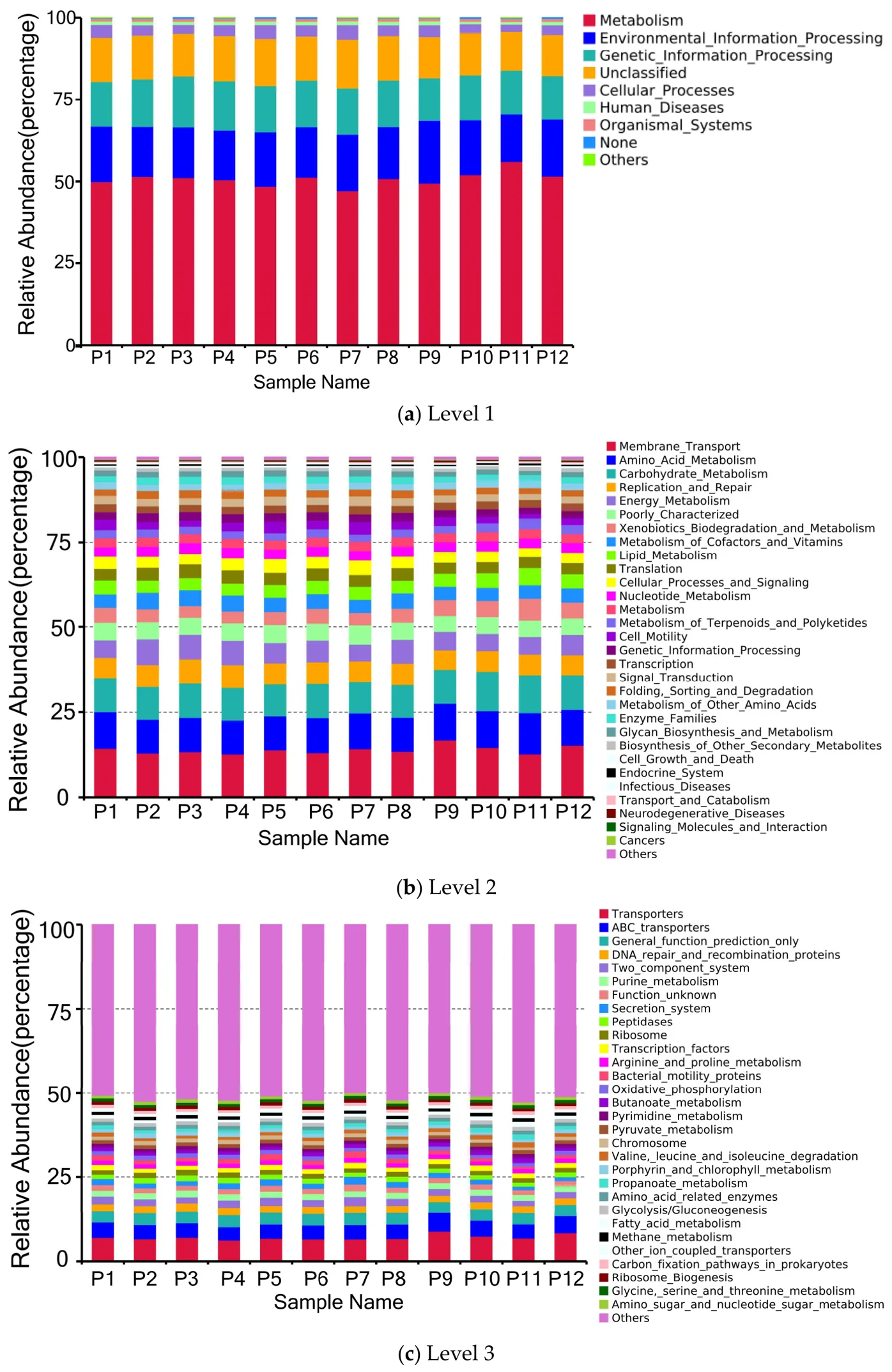
Relative abundance of PICRUSt-predicted bacterial functions in *H. rhamnoides* root samples from 12 sampling sites. (**a**) Level 1; (**b**) Level 2; (**c**) Level 3. Note: The data is the average value, *n* = 3.

PICRUSt was used to predict the functional potential of root endophytic bacterial communities from 12 sampling sites of *H. rhamnoides*, and a total of eight level-1 functions were identified (Figure 6a). Among them, the top five were Metabolism, Environmental Information Processing, Genetic Information Processing, Unclassified, and Cellular Processes. Among all functional categories, the metabolic function potential predominated across the 12 sampling sites of *H. rhamnoides*, with relative abundances ranging from 47.24% to 56.02%, peaking at P11 and reaching the minimum at P7. The potentials of environmental information processing, genetic information processing, and unclassified functions ranked next, with relative abundances ranging from 14.54% to 19.14%, 13.01% to 15.63%, and 11.89% to 14.96%, respectively. Further comparison revealed that, compared with sampling site P11, the other 11 sampling sites showed decreases in both metabolic and unclassified functional potential, while the potential for environmental information processing increased correspondingly; and compared with sampling site P9, the other 11 sampling sites exhibited decreases in environmental information processing potential, whereas the potential for genetic information processing showed an increasing trend.

Based on the PICRUSt-based prediction of level-2 functional potential (Figure 6b), a total of 30 functional modules were identified, among which 13 core functions (relative abundance > 75%) were predicted. The three functions with the highest predicted potential were Membrane_Transport (12.66–16.88%), Amino_Acid_Metabolism (9.98–12.15%), and Carbohydrate_Metabolism (9.16–11.58%), with a combined abundance exceeding 30%. Notably, compared with sampling site P9, the remaining 11 sampling sites were predicted to have lower functional potential for Membrane_Transport, but higher functional potential for Replication_and_Repair, Poorly_Characterized, and Translation.

Based on the PICRUSt-based prediction of level-3 functional potential (Figure 6c), a total of 30 functional modules were identified, among which 10 core functions (relative abundance > 25%) were predicted. The three functions with the highest predicted potential were Transporters (6.26–8.878%), ABC_transporters (3.91–5.56%), and General_function_prediction_only (3.24–3.62%), with a combined abundance exceeding 12%. Notably, compared with sampling site P9, the remaining 11 sampling sites were predicted to have lower functional potential for Transporters and ABC_transporters, but higher functional potential for General_function_prediction_ only.

### 3.7. Cluster Analysis of Endophytic Bacterial Communities in the Roots of H. rhamnoides from 12 Sampling Sites

Hierarchical clustering of the top 16 bacterial taxa based on phylum-level relative abundance was performed, with results presented in Supplementary Figure S4. At a clustering distance of 0.47, the 12 sampling sites were divided into two groups: sampling sites P6, P5, P9, P1, P7, P4, P2, P8, and P3 were assigned to Group 1, while sampling sites P12, P10, and P11 were assigned to Group 2. Further subdivision revealed that when the clustering distance decreased to 0.42, Group 1 could be divided into two subgroups, with sampling sites P6, P5, P9, P1, and P7 clustering into one subgroup, while the remaining sampling sites constituted the other subgroup. At a clustering distance of 0.16, Group 2 was similarly divided into two subgroups, with sampling site P11 forming a separate cluster, while P12 and P10 clustered into the other subgroup. Notably, the sampling sites in Group 1 were concentrated on the same phylogenetic branch in the Bray–Curtis hierarchical clustering dendrogram, indicating high similarity in bacterial community structure across these regions. In contrast, the sampling sites in Group 2 were distributed across different branches, suggesting marked differentiation in bacterial community composition.

### 3.8. The Coupled Relationship Between Climatic Characteristics, Soil Physicochemical Parameters, and the Diversity of Endophytic Bacterial Communities in H. rhamnoides Roots

#### 3.8.1. Mantel Test Analysis of Climatic Characteristics, Soil Physicochemical Parameters, and Endophytic Bacterial Communities in Roots

**Figure 7:**
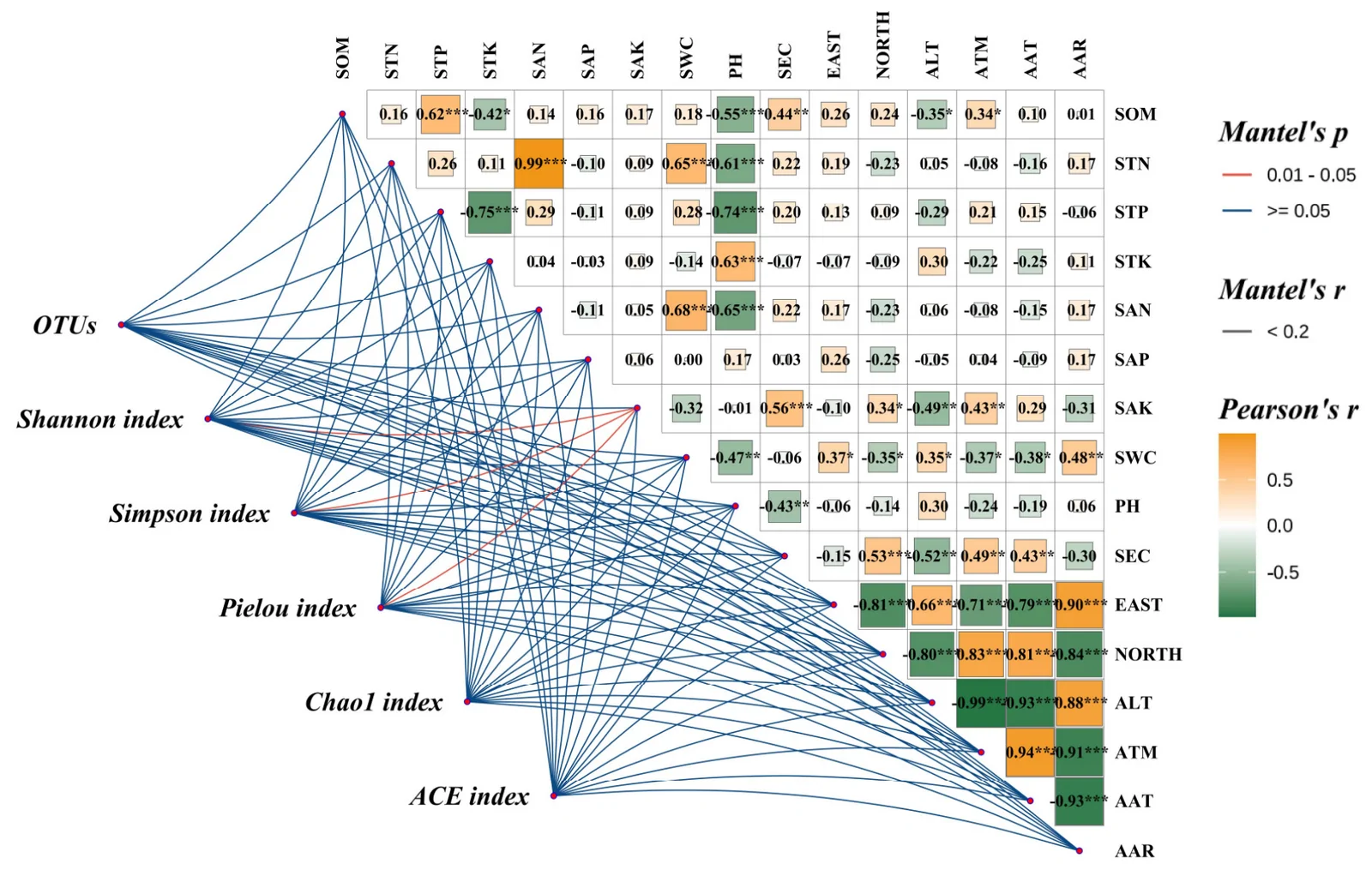
Climatic characteristics, physicochemical properties of soil, and Mantel analysis of root endophytic bacterial communities. Note: The independent sample size for environmental analysis is *n* = 12; orange is positively correlated, green is negatively correlated, and the darker the color, the stronger the correlation; * denotes *p* < 0.05, ** denotes *p* < 0.01, *** denotes *p* < 0.001.

The Mantel test results are presented in Figure 7. Regarding soil factors, SOM exhibited an extremely significant positive correlation with STP (*p* < 0.001) and an extremely significant negative correlation with pH (*p* < 0.001). STN exhibited extremely significant positive correlations with both SAN and SWC (*p* < 0.001) and an extremely significant negative correlation with pH (*p* < 0.001). STP showed extremely significant negative correlations with both pH and STK (*p* < 0.001). SAN exhibited an extremely significant positive correlation with SWC (*p* < 0.001) and an extremely significant negative correlation with pH (*p* < 0.001). Regarding geographic factors, EAST exhibited extremely significant positive correlations with both AAR and ALT (*p* < 0.001) and extremely significant negative correlations with AAT, ATM, and NORTH (*p* < 0.001). NORTH exhibited extremely significant positive correlations with both AAT and ATM (*p* < 0.001) and extremely significant negative correlations with both AAR and ALT (*p* < 0.001). ALT exhibited an extremely significant positive correlation with AAR (*p* < 0.001) and extremely significant negative correlations with both AAT and ATM (*p* < 0.001). ATM exhibited an extremely significant positive correlation with AAT (*p* < 0.001) and an extremely significant negative correlation with AAR (*p* < 0.001). Regarding the diversity indices of endophytic bacteria in the roots of *H. rhamnoides*, the Shannon, Simpson, and Pielou indices were all significantly correlated with SAK (*p* < 0.05), whereas the remaining three diversity indices showed no significant or highly significant correlations with climatic and soil factors.

#### 3.8.2. RDA Analysis of Climatic Characteristics, Soil Physicochemical Parameters, and Endophytic Bacterial Communities in Root Systems

**Figure 8:**
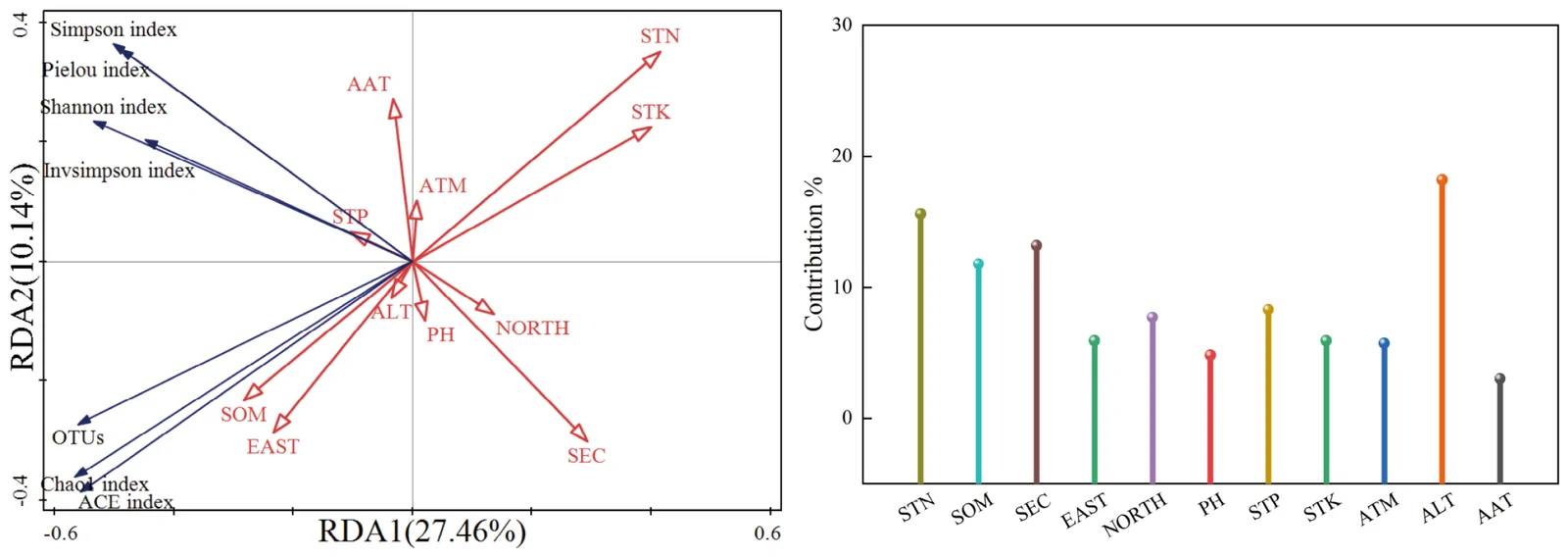
RDA analysis of climate characteristics, soil physicochemical properties, and root endophytic bacterial community structure. Note: The red arrows represent climatic characteristics, geographic information, and soil factors, while the blue arrows represent bacterial diversity indices. EAST: East longitude; NORTH: Northern latitude; ALT: Altitude; ATM: Air pressure; AAT: Average annual temperature; SOM: Soil organic matter; STN: Soil total nitrogen; STP: Soil total phosphorus; STK: Soil total potassium; PH: Soil pH; SEC: Soil electrical conductivity.

The redundancy analysis (RDA) results are presented in Figure 8, revealing the relationships between root endophytic bacterial community diversity and climatic as well as soil physicochemical factors in *H. rhamnoides*. The explanatory rates of the first two axes to the root endophytic bacterial community diversity were 27.46% and 10.14%, respectively, with a cumulative total of 37.60%. Among them, the arrow of ALT is the longest, with an explanation rate of 7.0% and a contribution rate of 18.2%, which has the most influence. STN followed, with an explanation rate of 6.0% and a contribution rate of 15.6%; it did not reach a significant level. The results indicated that ALT was the primary environmental factor driving the variation in root endophytic bacterial community diversity of *H. rhamnoides*, followed by STN as the second most influential factor.

## 4. Discussion

### 4.1. Changes in the Structure and Function of Endophytic Bacterial Communities in the Roots of H. rhamnoides from 12 Sampling Sites

Endophytic microorganisms in plant roots are associated with soil ecosystem functions; they exert a profound influence on the growth and development of host plants by accelerating organic matter decomposition, promoting nutrient cycling, and antagonizing pathogens [18]. In this study, 12 sampling sites of *H. rhamnoides* in the arid region of Northwest China were selected as research objects. Rhizosphere soil samples were systematically collected, and the combined approach of high-throughput amplicon sequencing and soil physicochemical property analysis was employed to observe and analyze the composition characteristics of root endophytic bacterial communities of *H. rhamnoides* and their associations with habitat factors. The observational data obtained in this study revealed that the soil physicochemical factors and geographical and climatic conditions at different *H. rhamnoides* sampling sites exhibited pronounced differentiation. Correspondingly, the composition and structural differentiation of the root endophytic bacterial communities in these sites also showed changes, which were indirectly associated with the growth indicators of *H. rhamnoides* plants. In all the observed plots, the number of endophytic bacterial ASVs detected in the roots of *H. rhamnoides* at sampling site P8 was the most abundant (over 400), far exceeding the observed value at sampling site P5 (fewer than 200). This observed distribution pattern is consistent with the ecological habit of *H. rhamnoides* adapting to alkaline, arid habitats documented in published studies [4]. Based on this, a hypothesis can be proposed: alkaline and arid soil environments may strengthen the mutualistic symbiosis between *H. rhamnoides* and its root endophytic bacteria, thereby indirectly promoting the expansion of the bacterial groups colonizing the root system [4,19]. It is noteworthy that, in habitats where the observed soil total nitrogen content was relatively low (e.g., sampling site P8), the ASV count of root endophytic bacteria of *H. rhamnoides* instead reached the highest level among all sampling sites. Based on the available indirect evidence, we propose a hypothetical causal relationship: insufficient soil nitrogen supply may act as a potential environmental driver, inducing the roots of *H. rhamnoides* to increase the secretion of enzymes and organic substances, thereby recruiting functional endophytic bacterial groups such as nitrogen-fixing bacteria and enhancing nitrogen availability in the rhizosphere microenvironment to meet the nutrient demands of the plant [20,21,22].

The root endophytic bacterial communities across 12 *H. rhamnoides* sampling sites in the Northwest Arid Region of China exhibited high consistency in dominant phylum composition. Proteobacteria, Actinobacteria, Cyanobacteria, Bacillota, and Bacteroidetes occupied the top five abundance positions within the root endophytic bacterial community, indicating that these phyla play irreplaceable functional roles in the root micro-ecosystem of *H. rhamnoides* [4]. However, the species abundance among various sampling sites shows differentiation characteristics, which are likely to be caused by the driven screening of environmental factors such as soil water content [23]. A comparison of the microbial community data from different sampling sites revealed that at sampling site P5, which had relatively higher water content, the abundance of Actinobacteria decreased, and the genera *Promicromonospora* and *Streptomyces* within this phylum also showed a reduction at the genus level. This result further validates the conclusion from the previous correlation analysis that the abundances of these taxa are correlated with soil water content. Further controlled experiments could be conducted to clarify the interaction between them. At the same time, the α-diversity of endophytic bacteria in the root systems of *H. rhamnoides* from 12 sampling sites also exhibited spatial heterogeneity. Compared with sampling site P5, the remaining 11 sampling sites (particularly P8) showed significantly higher ASV richness, Ace index, and Chao1 index values. This result is consistent with existing findings on the distribution patterns of rhizosphere microbial diversity in desert plants in arid regions [24,25]. Overall, this spatial variation in α-diversity is primarily attributed to the heterogeneity in soil physicochemical characteristics (including pH, moisture content, nutrient availability, etc.) and climatic conditions across different sampling sites [24,25].

The co-occurrence molecular network of root endophytic bacteria can visually reveal the interactions among bacterial taxa and the stability of the community [26,27]. Our results showed that Proteobacteria and Actinobacteriota constituted the core backbone of the endophytic bacterial network in the roots of *H. rhamnoides*, exhibiting the highest degree and topological importance within the network. They play an irreplaceable role in maintaining the structural and functional stability of the bacterial community and in optimizing the rhizosphere soil environment [26]. The endophytic bacterial network structure was highly complex, comprising 506 nodes and 1812 edges, with positive correlations predominating among the populations. This indicates a synergistic response between the endophytic bacteria in the roots of *H. rhamnoides* and the soil environment, and such a network structure facilitates efficient capture and utilization of nutrients by plant roots [27]. Cluster analysis revealed that the root endophytic bacterial communities from 12 sampling sites of *H. rhamnoides* on the Qinghai–Tibet Plateau could be divided into two major groups: Group 2 comprised only three sampling sites (P12, P10, and P11), while Group 1 contained the remaining nine sampling sites. This grouping pattern may be related to differences in vegetation and soil properties among the sampling sites, and climatic factors such as altitude, temperature, and precipitation may also have exerted some influence [28,29].

Based on the functional potential of bacteria predicted by PICRUSt software, the root endophytic bacterial communities of *H. rhamnoides* from the 12 sampling sites exhibited a certain degree of differentiation in functional potential, which is consistent with the conclusion in existing studies that “community structure drives differences in functional potential" [25,26]. At the level of specific functional potential, metabolic functional potential predominated across all sampling sites, followed successively by environmental information processing, genetic information processing, and unclassified functions. As the basic metabolic framework for bacterial life activities, these functional potentials, by ensuring energy supply and material transformation, occupy a core ecological niche in the endophytic bacteria of *H. rhamnoides* roots, thereby supporting the stable functioning of the soil system [4,30,31,32]. In addition, the genetic information processing functional potential at sampling site P9 was significantly weaker than that at the other sampling sites, which may be attributed to the harsher habitat conditions at this site. The nutrient-poor soil environment, by restricting resource availability, affects the normal life activities of the bacterial communities, thereby constraining the growth and reproduction of the endophytic bacterial communities in the roots of *H. rhamnoides* [33,34,35].

### 4.2. Analysis of the Correlation Between the Diversity of Endophytic Bacterial Communities in the Root Systems of H. rhamnoides and Soil Physicochemical Properties and Climatic Characteristics

The results of the Mantel test revealed a significant correlation between the physicochemical properties of the soil in the root zone of *H. rhamnoides* (including organic matter, total nitrogen, total phosphorus, total potassium, pH, and electrical conductivity) and the climatic characteristics of the sampling sites (longitude, latitude, elevation, precipitation, and temperature). Specifically, soil organic matter (SOM), total nitrogen (STN), and total phosphorus (STP) were all significantly and negatively correlated with pH, which may be attributed to the inhibition of organic matter decomposition and nitrogen–phosphorus mineralization under alkaline conditions [36]. Total nitrogen (STN) and alkali-hydrolyzable nitrogen (SAN) both exhibited extremely significant positive correlations with soil water content (SWC). This pattern is presumably attributed to their close association with microbial metabolic activity—moderately moist soil conditions favor microbial proliferation and enhanced enzymatic activity, thereby accelerating the transformation of organic matter into inorganic nitrogen [37,38]. In addition, SOM shows a significant negative correlation with elevation (ALT), which may stem from dual constraints on microbial activity and vegetation growth: high elevation weakens the metabolic activity of rhizosphere microorganisms while simultaneously inhibiting the normal growth and development of *H. rhamnoides* plants, ultimately leading to reduced accumulation of soil organic matter [39,40]. Further analysis indicated that there were also significant associations between bacterial community diversity indices and soil physicochemical properties and climatic factors; specifically, diversity indices such as the Shannon index, Pielou index, and Simpson index all showed significant correlations (*p* < 0.05) with soil physicochemical properties (SAK). These results indicate that *H. rhamnoides* natural populations on the Qinghai–Tibet Plateau achieve a bidirectional driving effect—enhancing soil fertility while simultaneously optimizing bacterial community structure—through the synergistic interaction among vegetation, soil, and microorganisms [41,42,43].

Soil physicochemical properties and climatic conditions exert significant shaping effects on the diversity and structure of root endophytic bacterial communities in *H. rhamnoides* on the Qinghai–Tibet Plateau, with altitude (ALT) playing a particularly pivotal regulatory role. Field survey results revealed that the altitudinal gradient showed a partial correlation with the α-diversity of endophytic bacterial communities in the roots of *H. rhamnoides*. In combination with relevant conclusions reported in the literature [44,45,46,47], the following possible pathways of association can be identified. First, high-altitude areas are often accompanied by relatively low temperatures, and existing studies have demonstrated a clear correlation between low-temperature habitat conditions and reduced bacterial metabolic activity [44,45]. Second, the harsh environmental conditions at high altitudes are associated with constrained vegetation productivity and reduced litter return, which in turn affect soil nutrient parameters; such changes in nutrient factors are correlated with the growth characteristics of bacterial communities [46,47]. Among the factors influencing bacterial community diversity, soil total nitrogen (STN) ranked second only to altitude in terms of effect strength, primarily reshaping bacterial community structure through direct nutrient stress effects. This conclusion is similar to existing findings on the driving mechanisms of the plant root microbial environment in Northeast China [48].

Based on the observational foundation of this study, future research will include multiple batches of independent biological replicate sampling at the 12 target *H. rhamnoides* sampling sites. Concurrently, habitat factors that were not previously taken into account, such as climatic fluctuations in the sampling years and micro-topographic characteristics, will be recorded. Through a rigorous block experimental design, the statistical support will be strengthened. Due to the limitation of the 515F/806R primer pair used in this study, which exhibits a bias toward amplifying plant organellar sequences, we will completely switch to the specific primer pair 799F/1193R for subsequent isolation, identification, and sequencing analysis of endophytic bacteria in plant roots. In addition, a peptide nucleic acid (PNA) clamp targeting chloroplast sequences will be incorporated into the PCR amplification system to block the non-specific enrichment of host chloroplast sequences at the amplification source. This approach will thoroughly eliminate interference from such contamination in the analysis of endophytic bacterial communities, thereby establishing a sequencing system with lower background and higher accuracy for future studies. Furthermore, subsequent studies will also supplement biochemical and physiological verification experiments, including in situ determination of root physiological indicators and detection of key enzyme activities in rhizosphere soil, combined with controlled inoculation interaction experiments between *H. rhamnoides* and root endophytic bacteria. Targeted verification will be conducted on all model-derived results obtained in this study, such as taxonomic annotation based on high-throughput sequencing, PICRUSt functional prediction, and community clustering analysis, to clarify the potential causal boundaries of the microbiota–environment association hypotheses proposed in this study. This will ultimately provide a solid foundation, with both statistical repeatability and direct experimental evidence support, for rigorously elucidating the adaptive mechanisms of the symbiotic system between *H. rhamnoides* and its root endophytic bacteria in the arid region of Northwest China.

## 5. Conclusions

In this study, *H. rhamnoides* in the arid region of Northwest China was used as the plant material to analyze the community composition of its root endophytic bacteria and their correlations with environmental factors. The main conclusions are as follows: (1) The number of bacterial ASVs varied markedly among the 12 sampling sites, with sampling site P8 having the highest number (435), approximately 2.8 times that of sampling site P5 (156); in terms of bacterial alpha diversity indices, sampling site P8 ranked highest in both the Ace and Chao1 indices, while sampling site P5 ranked the lowest. (2) Proteobacteria, Actinobacteria, Cyanobacteria, Bacillota, and Bacteroidetes were the dominant groups in the bacterial community. The relative abundance of Proteobacteria exceeded 18% at all sampling sites, accounting for the highest proportion. (3) PICRUSt functional prediction showed that the predicted functional potential of Metabolism, Environmental Information Processing, and Genetic Information Processing accounted for relatively high proportions at all sampling sites. Among them, sampling site P9 exhibited the strongest predicted potential in Environmental Information Processing, while its predicted potential in Genetic Information Processing was relatively weak. Bray–Curtis clustering divided the 12 sampling sites into two groups: sampling sites P12, P10, and P11 clustered into one group, and the remaining nine sampling sites formed the other group. (4) Redundancy analysis (RDA) indicated that altitude (ALT) and soil total nitrogen (STN), through the synergistic effects of climatic factors and soil physicochemical factors, jointly shaped the alpha diversity and the abundance distribution patterns at the phylum and genus levels of the root endophytic bacterial community.

In summary, habitat heterogeneity, including soil and climatic factors, may shape differentiated root microenvironments by influencing the growth of *H. rhamnoides*, thereby driving adaptive differentiation of the bacterial community; at the same time, certain correlations exist between the characteristics of the root bacterial community of *H. rhamnoides* and soil–climate factors. Future research will focus on the isolation and identification of endophytic bacteria from *H. rhamnoides*, verify the growth-promoting effects of the strains through inoculation experiments, analyze changes in the medicinal components of *H. rhamnoides* using metabolomics, and evaluate the potential applicability of these strains in other alpine plants.

## Data Availability

The original data presented in the study are openly available in NCBI at https://www.ncbi.nlm.nih.gov/sra/PRJNA (accessed on 5 August 2026), reference number 1507603. The others in this study are included in the article/Supplementary Material. Further inquiries can be directed to the corresponding author.

## Supplementary Materials

The following supporting information can be downloaded at: https://www.mdpi.com/article/10.3390/microorganisms14081829/s1, Figure S1: Alpha diversity of bacterial communities from 12 sampling sites (Invsimpson index, Chao1 index, ACE index, Goods coverage); Figure S2: Differences in β-diversity (PCoA) of bacterial communities from 12 sampling sites; Figure S3: Co-occurrence network of root endophytic bacterial communities across 12 sampling sites; Figure S4: Bray–Curtis hierarchical clustering dendrogram; Table S1: Geographic information and climatic characteristics of *H. rhamnoides* sampling sites; Table S2: Physicochemical properties of soils in *H. rhamnoides* distribution areas.
