# Supplementary material for: Geographic Variation Characteristics of Endophytic Bacterial Communities in Roots of Hippophae rhamnoides subsp. sinensis Rousi in the Arid Region of Northwest China
Source: Microorganisms. 2026 Aug 19;14(8):1829. doi: 10.3390/microorganisms14081829 (PMC13515303; doi:10.3390/microorganisms14081829)
Supplement: Supplementary file 1 [file microorganisms-14-01829-s001.zip › microorganisms-4445038-supplementary.pdf]

microorganisms

Supplementary Material

1 Supplementary Table

Supplementary Table S1. Geographic information and climatic characteristics of *H. rhamnoides* sampling sites. **Note:** EAST: East longitude; NORTH: Northern latitude; ALT: Altitude; ATM: Air pressure; AAT: Average annual temperature; AAR: Average annual rainfal.

Serial number	Sampling sites	EAST/°	NORTH/°	ALT/m	ATM/KPa	AAT/℃	AAR/mm
P1	Ping'an District	102.11	36.50	2497.83	73.83	3.78	114.90
P2	Xunhua Salar Autonomous County	102.49	35.85	2610.68	74.07	2.38	121.89
P3	Hualong Huizu Autonomous County	102.26	36.09	2503.98	73.09	3.67	114.75
P4	Menyuan Huizu Autonomous County	101.61	37.39	3369.01	67.98	−1.77	134.44
P5	Maqin County	100.24	34.48	3353.58	67.94	0.62	125.88
P6	Datong Huzui and Tuzu Autonomous County	101.69	36.93	2748.76	73.24	2.60	125.64
P7	Huangzhong District	101.57	36.50	2906.80	71.94	1.86	124.36
P8	Guinan County	100.75	35.59	3522.05	66.80	0.25	120.74
P9	Tongde County	100.58	35.25	3369.01	67.94	1.47	124.87
P10	Minle County	100.81	38.43	2692.50	73.17	0.88	116.38
P11	Yongdeng County	103.26	36.74	2214.63	77.87	4.39	101.47
P12	Hejing County	86.38	42.32	1106.30	87.81	10.76	31.56

Supplementary Table S2. Physicochemical properties of soils in *H. rhamnoides* distribution areas. **Note:** SOM: Soil organic matter; STN: Soil total nitrogen; STP: Soil total phosphorus; STK: Soil total potassium; SAN: Soil alkali-hydrolyzable nitrogen; SAP: Soil available phosphorus; SAK: Soil available potassium; SWC: Soil water content; SEC: Soil electrical conductivity.

**Serial numb er**	**SOM/ (g/kg)**	**STN/ (g/kg)**	**STP/ (g/kg)**	**STK/ (g/kg)**	**SAN/ (mg/k**	**SAP/ (mg/k**	**SAK/ (mg/k**	**SWC/ (%)**	**pH**	**SEC/ (ms/c**
P1	44.27	2.65	1.46	13.98	199.85	0.79	19.01	17.73	6.85	0.56
P2	32.45	0.85	0.61	18.20	44.85	17.45	54.29	11.78	8.28	0.56
P3	9.59	1.98	0.68	17.66	136.58	3.97	152.80	12.27	7.97	0.60
P4	22.78	1.32	0.52	19.34	89.09	0.93	21.39	18.19	8.08	0.61
P5	3.86	5.19	0.57	20.63	373.02	3.31	33.42	22.01	7.75	0.53
P6	30.93	3.09	0.51	19.51	213.33	3.16	66.06	12.29	7.48	0.62
P7	31.18	1.91	0.62	18.26	124.93	2.09	28.36	19.76	8.01	0.48
P8	3.65	0.48	0.37	18.37	21.18	0.51	18.44	7.81	8.36	0.50
P9	3.60	0.61	0.55	20.68	18.01	0.64	36.40	7.62	8.72	0.48
P10	43.62	2.54	0.92	19.44	158.57	1.18	135.01	12.83	7.84	0.60
P11	44.50	2.63	0.72	19.22	158.82	2.83	89.63	10.70	7.92	0.60

P12	16.13	1.12	0.61	18.58	70.91	0.37	84.83	6.95	7.95	0.60

2 Supplementary Figures

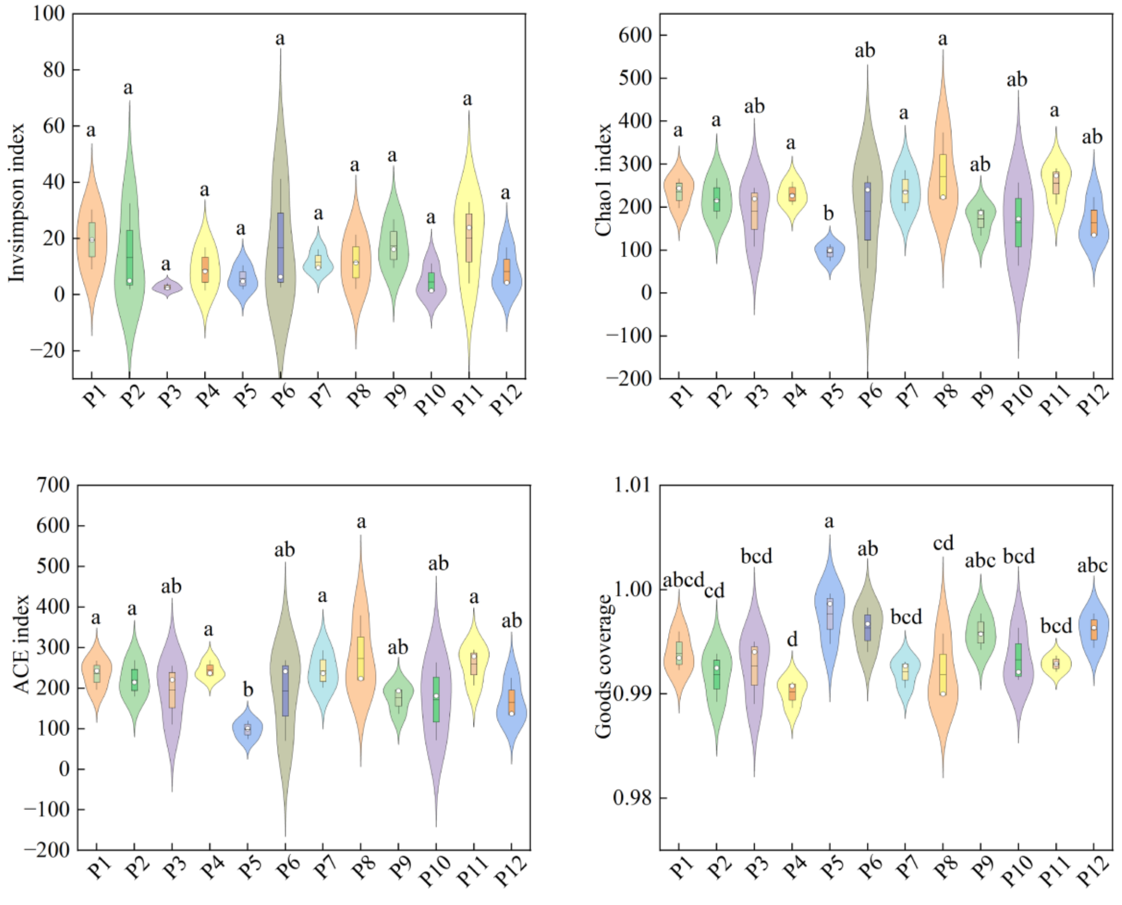

Supplementary Figure S1. Alpha diversity of bacterial communities from 12 sampling sites (Invsimpson index, Chao1 index, ACE index, Goods coverage). **Note:** Different lowercase letters indicate significant differences at the $p < 0.05$ level.

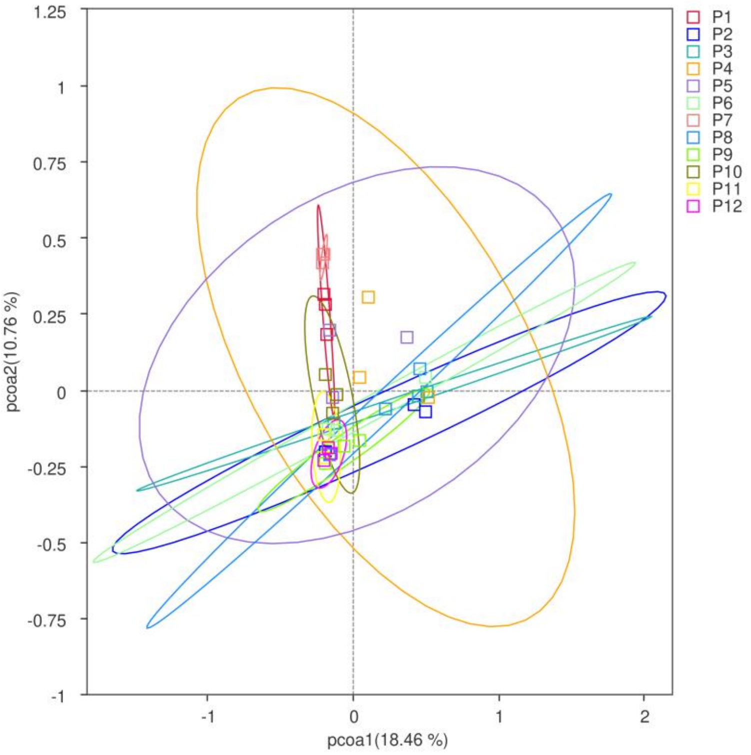

Supplementary Figure S2. Differences in β-diversity (PCoA) of bacterial communities from 12 sampling sites.

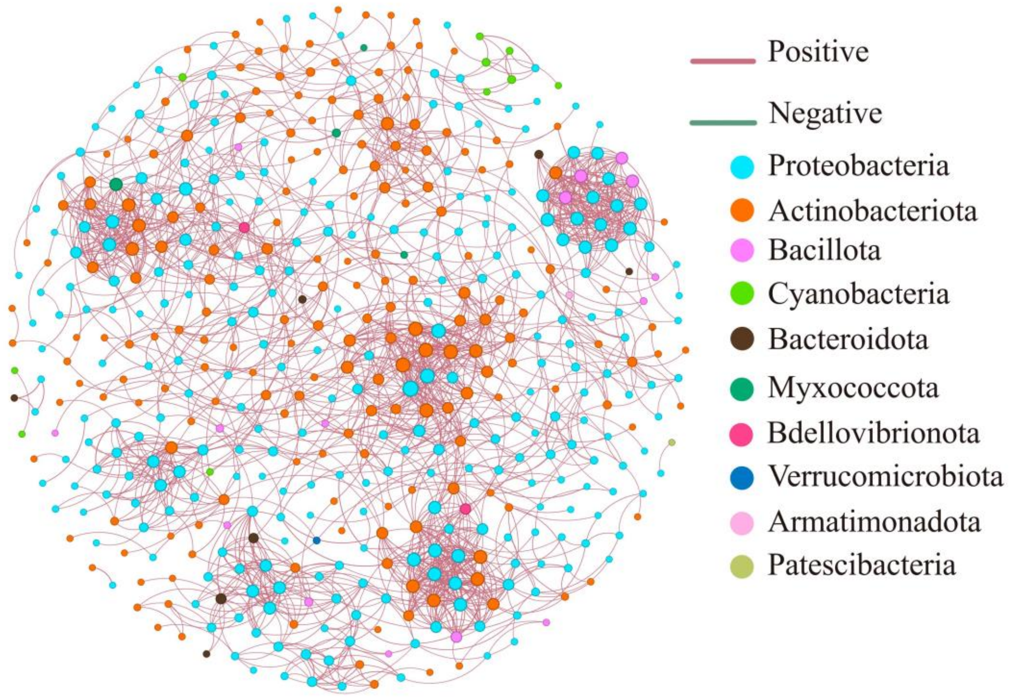

Supplementary Figure S3. Co-occurrence network of root endophytic bacterial communities across 12 sampling sites. **Note:** Node colours and sizes indicate species type and importance.

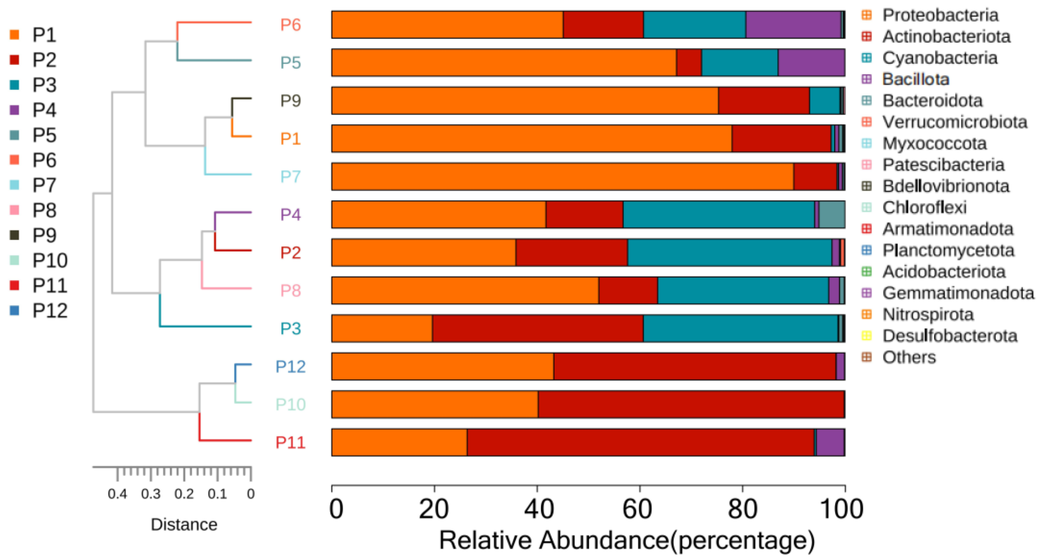

Supplementary Figure S4. Bray–Curtis hierarchical clustering dendrogram. **Note:** Left panel illustrates the UPGMA clustering tree structure; right panel presents the phylum-level relative abundance profile of soil bacteria.
